# A Novel Silver Bioactive Glass Elicits Antimicrobial Efficacy Against *Pseudomonas aeruginosa* and *Staphylococcus aureus* in an *ex Vivo* Skin Wound Biofilm Model

**DOI:** 10.3389/fmicb.2018.01450

**Published:** 2018-07-03

**Authors:** Holly N. Wilkinson, Sammi Iveson, Paul Catherall, Matthew J. Hardman

**Affiliations:** ^1^School of Life Sciences, University of Hull, Hull, United Kingdom; ^2^Theraglass Ltd., London, United Kingdom

**Keywords:** biofilm, bioactive glass, wound healing, *Pseudomonas aeruginosa*, *Staphylococcus aureus*

## Abstract

Biofilm infection is now understood to be a potent contributor to the recalcitrant nature of chronic wounds. Bacterial biofilms evade the host immune response and show increased resistance to antibiotics. Along with improvements in antibiotic stewardship, effective new anti-biofilm therapies are urgently needed for effective wound management. Previous studies have shown that bioactive glass (Bg) is able to promote healing with moderate bactericidal activity. Here we tested the antimicrobial efficacy of a novel BG incorporating silver (Bg^Ag^), against both planktonic and biofilm forms of the wound-relevant bacteria *Pseudomonas aeruginosa* and *Staphylococcus aureus*. Bg^Ag^ was stable, long lasting, and potently effective against planktonic bacteria in time-kill assays (6-log reduction in bacterial viability within 2 h) and in agar diffusion assays. Bg^Ag^ reduced bacterial load in a physiologically relevant *ex vivo* porcine wound biofilm model; *P. aeruginosa* (2-log reduction) and *S. aureus* (3-log reduction). Bg^Ag^ also conferred strong effects against *P. aeruginosa* biofilm virulence, reducing both protease activity and virulence gene expression. Co-culture biofilms appeared more resistant to Bg^Ag^, where a selective reduction in *S. aureus* was observed. Finally, Bg^Ag^ was shown to benefit the host response to biofilm infection, directly reducing host tissue cell death. Taken together, the findings provide evidence that Bg^Ag^ elicits potent antimicrobial effects against planktonic and single-species biofilms, with beneficial effects on the host tissue response. Further investigations are required to elucidate the specific consequences of BG administration on polymicrobial biofilms, and further explore the effects on host–microbe interactions.

## Introduction

Breaches to the skin barrier must be repaired in a timely and efficient manner to prevent infection and wound persistence (Bjarnsholt et al., 2008). Unfortunately, healing often fails, leading to chronic, non-healing wounds. Chronic wounds affect millions of patients annually, with associated substantial costs to healthcare providers (Posnett and Franks, 2008; Gould et al., 2015). In recent years, one of the most discussed extrinsic contributors to wound chronicity has been the presence of infection, particularly critical colonization by pathogenic organisms existing as biofilms (Dow et al., 1999; James et al., 2008; Wolcott et al., 2015). Biofilms are bacterial aggregates able to evade host responses and develop rapid pathogenicity (reviewed in Gellatly and Hancock, 2013; Flemming et al., 2016). They are encapsulated in self-produced extracellular polymeric substances (EPS), which provide a protective barrier against both host defenses and exogenous antibiotic treatments (Flemming and Wingender, 2010). Moreover, biofilms consist of heterogeneous communities which allows inter-specific transfer of metabolites and antimicrobial-resistance genes, thus increasing overall bacterial virulence (Leid et al., 2005; Mah, 2012). Sadly, wound infection is on the rise, with the prevalence of antibiotic-resistant bacterial strains and the persistence of biofilms contributing to the recalcitrant nature of chronic wounds (Velnar et al., 2009). Antibiotic resistance is not only a problem for wound management but a widespread threat to human health, claiming in excess of 50,000 lives in western society alone (O’Neill, 2014). Consequently, the implementation of antibiotic stewardship programs worldwide aim to prevent misuse thus reducing antibiotic resistance and improving patient outcome (Bowler, 2018).

Bioactive materials are a concept first introduced by L. L. Hench, who stated that the main requirement of a bioactive material was to elicit a biological response at the interface between the material and the living tissue (Hench, 2006). Bioactive glass (BG) has emerged as one such bioactive material, which has the potential to promote dental and bone repair. BG compositions can include compounds with known pro-healing effects (e.g., CaO, SiO_2_, P_2_O_5_, and MgO; Lin et al., 2012). BGs bond compatibly to tissues via dissolution and precipitation reactions that actuate surface ion exchange. In damaged bone tissue, BG ion release increases pH, causing the formation of a hydroxycarbonate apatite (HCA) interface layer, which mimics the type of scaffold that would naturally form during tissue repair (Hench, 2006). This HCA interface then promotes cell migration, osteoprogenitor differentiation (Jones, 2013; Miguez-Pacheco et al., 2015) and ultimately accelerates bone matrix reformation (as assessed in Maçon et al., 2017). Interestingly, elevated alkalinity can provide a bactericidal environment, thus BG alone may have antibacterial effects (Zehnder et al., 2006). BGs can also be used as a substrate for incorporating known antimicrobials, such as metal nanoparticles (Gholipourmalekabadi et al., 2016).

Silver (Ag) and silver nanoparticles (AgNPs) exhibit potent antimicrobial efficacy against a range of bacterial species (Ruparelia et al., 2008), including antibiotic-resistant strains (Mekkawy et al., 2017; Tiwari et al., 2017a,b) and established biofilms (Ueno et al., 2016) in experimental settings. In the clinic, the evidence for silver efficacy is less robust, underpinning variability in clinical use (Rodriguez-Arguello et al., 2018). Incorporating Ag and AgNPs into BG has been suggested as a potential alternative to antibiotics for reducing infection in a range of clinical applications (Zhao et al., 2015). Theraglass^TM^ (Theraglass Ltd., United Kingdom) is a novel, highly bioactive, binary (SiO_2_ and CaO) sol–gel derived BG (Saravanapavan and Hench, 2001; Bellantone et al., 2002; Poologasundarampillai et al., 2014; Maçon et al., 2015). Advantages of sol–gel derived BG over more traditional melt-derived BG include easier manufacturing processes and higher purity (Saravanapavan and Hench, 2001). Theraglass^TM^ also exhibits a larger surface area to volume ratio than other BG formulations, which may convey higher overall bioactivity (Maçon et al., 2015). One formulation of Theraglass^TM^ also incorporates silver (AgO) hence it maintains high potential as an antimicrobial therapeutic.

In the present study, we aimed to elucidate the antimicrobial and anti-biofilm efficacy of a novel, highly bioactive, binary sol–gel derived AgO-functionalized BG (Theraglass^TM^) against the opportunistic wound pathogens *Pseudomonas aeruginosa* and *Staphylococcus aureus*.

## Materials and Methods

### Bioactive Glass Formulations

The elemental composition of Theraglass^TM^ by mol% is 70 SiO_2_:30 CaO, while Theraglass^TM^ Ag contains 70 SiO_2_:28 Ca:2 AgO by mol%. Topical treatments were prepared from powders containing BG (Theraglass^TM^; “Bg”) and BG containing silver (Theraglass^TM^ Ag; “Bg^Ag^”), mixed with a vehicle (Nivea, Beiersdorf) to produce BG preparations at a 20% (w/v) working concentration. A 2% (w/v) silver (AgO) control (“Ag”) was included, at the equimolar concentration of AgO in Bg^Ag^, by mixing the vehicle with AgO. For all experiments, except stability and kinetics testing, treatments were prepared and applied fresh (i.e., immediately post-mixing).

### Bacterial Cell Culture

Clinical reference strains of *P. aeruginosa* (NCTC 10781) and *S. aureus* (NCTC 13297) were chosen in this study because of their direct relevance in chronic wound infection (Price et al., 2009). Bacterial stocks stored at -80°C in glycerol were streaked onto Mueller–Hinton agar (MHA; Oxoid) plates and incubated for 24 h at 37°C to produce viable colonies. Overnight (O/N) cultures were prepared by incubating a single bacterial colony in 10 ml Mueller–Hinton broth (MHB; Oxoid) for 16 h at 37°C with 140 rpm shaking (Labnet 211DS shaking incubator, Labnet International). For all assays, O/N cultures were adjusted to 0.5 on the McFarland turbidity scale as described in Balouiri et al. (2016) unless otherwise stated. Briefly, bacterial cultures were diluted in 0.85% (w/v) sterile saline and adjusted to an optical density (OD) of 0.08–0.12 at 625 nm using a spectrophotometer (Jenway 7310, Cole-Parmer). Adjusted bacterial suspensions were further diluted 1:150 in MHB to give starting concentrations between 5 × 10^5^ and 1 × 10^6^ colony forming units per ml (CFU/ml), confirmed by spread plating on MHA.

### Agar Diffusion Testing

*Pseudomonas aeruginosa* and *S. aureus*, adjusted to the turbidity scale described above, were spread onto MHA (200 μl per plate). Sterile 1 cm^2^ pieces of Tegaderm^TM^ pad (3M) were used to apply treatments (described above) to MHA plates. A vehicle control (Tegaderm^TM^ Pad and Nivea) was also included. Treatments (*n* = 3 per group) were incubated at 37°C for 24 and 48 h. At the point of collection, plates were imaged with a Nikon camera (Finepix S5700, Nikon) and growth inhibition zones were measured in ImageJ v.1.6 (National Institutes of Health, NIH). Agar diffusion tests were repeated in three independent experiments.

### Time-Kill Assays

Adjusted cultures of *P. aeruginosa* and *S. aureus* in MHB were added to sterile universal tubes (5 ml per tube) with 1 g of fresh cream treatment (described above). Tubes were incubated at 37°C with 140 rpm shaking for 0.5, 1, 2, 3, 6, and 24-h time-points. Upon collection, 100 μl of bacterial broth was removed from each tube and added to 900 μl Dey-Engley Neutralizing broth (DENB; Fluka, Sigma-Aldrich). The DENB was tested prior to use to ensure it neutralized the antimicrobial treatments under study (data not shown). Samples were serial diluted, plated on MHA in duplicate, and incubated for 24 h at 37°C to determine CFU/ml. The time-kill assay was repeated in three independent experiments.

### Stability and Kinetics Testing

To test the stability of Ag, Bg, and Bg^Ag^, preparations (described above) were incubated at 4°C and room temperature (RT) for 24–120 h. At each 24-h interval, treatments were collected for agar diffusion assays against *P. aeruginosa* and *S. aureus* (as above). To test the kinetics of antimicrobial soluble factor release, Bg and Bg^Ag^ (1 g per tube) were incubated in tubes containing 5 ml of Dulbecco’s phosphate buffered saline (DPBS; Gibco, United Kingdom) at RT on a rocker over a period of 24–144 h, and collected at 24-h intervals. At each time point, supernatant (SN) was collected and replaced with fresh DPBS. Tegaderm^TM^ Pad was soaked in collected SN and added to bacterial plates for agar diffusion testing.

### Biofilm Formation Tests

Following our investigations of the antimicrobial efficacy of BG against planktonic bacteria, we tested the ability of BG to inhibit biofilm formation. Here, a standard 96-well microtiter plate assay was performed (as described in O’Toole, 2011). Briefly, 50 μl of MHB was added to each well, with treatments serial diluted (twofold dilutions) down each row. *P. aeruginosa, S. aureus*, and co-cultures (50:50 mix of *P. aeruginosa*:*S. aureus*), adjusted to the 0.5 McFarland standard and diluted as above, were added to each well (50 μl per well). Plates were incubated under aerobic conditions at 37°C for 48 h. After this time, plates were submerged in dH_2_O to remove planktonic bacteria, and 125 μl 0.1% (w/v) crystal violet was added to each well for 30 min to stain biofilms. Plates were washed in dH_2_O and dried O/N. To solubilize the crystal violet, 200 μl of 30% acetic acid was added to each well and plate absorbance was measured at 570 nm. Biofilm formation was categorized as in Christensen (1989) based on the ODs obtained above (see **Table 1**). The OD cut-off (ODc) was deduced as three standard deviations above the mean OD of the negative control. Biofilms were then classified as follows:

**Table 1:** Optical density classifications used to determine strength of biofilm production in biofilm formation assays.

OD_495 nm_ value	Classification
≤ 0.04	Non-adherent
>0.04 and ≤0.08	Weak biofilm
>0.08 and ≤0.16	Moderate biofilm
>0.16	Strong biofilm

OD ≤ ODc = non-adherent
ODc < OD ≤ 2 × ODc = weak biofilm
2 × ODc < OD ≤ 4 × ODc = moderate biofilm
4 × ODc < OD = strong biofilm

### *Ex Vivo* Porcine Wound Biofilm Model

To test the effects of BG on established biofilms, an *ex vivo* porcine wound biofilm model was employed (as in Wilkinson et al., 2016). Adjusted bacteria were added in 20 μl droplets to sterile nylon filter membranes (Merck-Millipore) on MHA and biofilms were left to form at the air-filter interface for 72 h at 37°C. Every 24 h, membrane biofilms were transferred to fresh MHA plates. For co-culture experiments, planktonic cultures of *P. aeruginosa* and *S. aureus* were mixed in a 1:1 ratio and added to filter membranes as above. Next, fresh porcine skin, collected under University ethical approval (FEC_21_2017) from a local abattoir, was held in high glucose Dulbecco’s Modified Eagle’s Medium (HG DMEM, Gibco) containing 10% (v/v) fetal bovine serum (FBS; Gibco), 100 U/ml penicillin–streptomycin (Gibco), 10 μg/ml gentamicin and 2.5 μg/ml Amphotericin B (both Sigma-Aldrich). The subcutaneous adipose tissue was removed; the skin was washed in Hank’s Balanced Salt Solution (HBSS, Sigma-Aldrich) containing 2× Antibiotic–Antimycotic (Gibco), and washed in DPBS prior to wounding.

The porcine skin was cut into 1 cm^2^ squares; wounds were created by complete removal of the epidermis, and were treated with established biofilms. Absorbent pads were soaked in HG DMEM containing supplements (as above). A filter membrane was placed between the absorbent pads and each porcine biofilm explant to allow nutrient transfer to the skin without submersion (modified from Stojadinovic et al., 2013). The skin was cultured at 37°C and 5% CO_2_ for 24 h to allow biofilm attachment. Treatments were then added to each biofilm (as described above), including non-treated biofilm controls and uninoculated porcine wound controls.

### Porcine Wound Biofilm Collection

Following 24 h of incubation, porcine *ex vivo* biofilm explants were bisected at their midpoint, with half wounds collected for viability counts (CFU/ml), histological analysis, extracellular protease production (via a colorimetric assay and zymography), and virulence factor expression.

#### Viability Counts

Tissue was cut into pieces (<1 mm) and vortexed in tubes containing 1 ml MHB and 5 ml sterile borosilicate glass beads (Sigma-Aldrich) for 3 × 10 s pulses. From each tube, 100 μl of re-suspended bacteria was removed and serial diluted in 900 μl DENB, with 100 μl of each dilution spread on MHA plates (for single species enumeration) or Cetrimide agar (Oxoid) and Mannitol Salt agar (Oxoid) for co-culture colony counts. Plates were incubated at 37°C for 24 h. Colonies were enumerated to give CFU/sample.

#### Biofilm Visualization

Samples embedded in optimal cutting temperature media (OCT, CellPath) were cryo-sectioned at 10 μm. Gram-Twort, Acridine Orange (AO; Sigma-Aldrich), and Concanavalin A (ConA, Thermo Fisher Scientific) staining were used to visualize porcine wound biofilm load. A modified Gram-Twort stain was carried out. Here, sections were fixed in methanol at -20°C for 10 min, stained with Crystal Violet and Gram’s Iodine solutions (both Sigma-Aldrich), differentiated in 2% (v/v) acetic-alcohol and counterstained with a 0.2% (w/v) neutral red and 0.2% (w/v) fast green (9:1) solution (Sigma-Aldrich). Sections were differentiated again, rapidly dehydrated in 100% ethanol and mounted with Pertex^®^ mounting medium (CellPath). Images were captured at ×100 magnification on a Nikon E400 microscope with SPOT camera (SPOT imaging). For fluorescent visualization, methanol-fixed sections were incubated in AO solution (2 mg/ml) for 5 min at RT and rinsed in dH_2_O, or incubated in ConA (50 μg/ml) at 4°C O/N and counterstained with DAPI (Thermo Fisher Scientific). Mowiol 4-88 (Sigma-Aldrich) containing DABCO (Thermo Fisher Scientific) was used for mounting. Fluorescent images were taken on a Zeiss Axio Vert. A1 microscope with AxioCam| cm1 camera (Carl Zeiss Microscopy Ltd) at ×40 magnification. Gram-Twort biofilm thickness analysis was performed in ImageJ v.1.6 (NIH).

#### Zymography

Bacteria isolated from treated porcine biofilms via glass bead dissociation (described above) were centrifuged at 10,000 × *g* and 4°C for 10 min. The SN fraction (containing extracellular proteases) was removed, sterile-filtered (0.22 μm, Merck-Millipore) and stored at -80°C until use. Gelatin zymography was performed to assess extracellular protease activity. SN was run on 7.5% acrylamide gels containing 0.2% (w/v) porcine skin gelatin (Oxoid) in non-reducing conditions. Precision Plus Protein^TM^ Kaleidoscope^TM^ Prestained Protein Standards (Bio-Rad) were used to determine molecular weights of the separated proteins in the gel. An MMP9 standard (Thermo Fisher Scientific) was included as an internal control. Gels, washed in 2.5% (v/v) Triton X-100, and incubated in gelatinase resolving buffer [1% (v/v) Triton X-100, 50 mM Tris–HCl, 5 mM CaCL_2_, and 1 μm ZnCl_2_ in dH_2_O with 0.02% (w/v) NaN_3_] for 24 h at 37°C, were stained with 0.2% (w/v) amido black (Thermo Fisher Scientific) and de-stained with 30% (v/v) acetic acid. Densitometric analysis of gels was performed in ImageJ v.1.6 (NIH) and relative protease expression determined.

#### Colorimetric Protease Detection

The Azocasein method (Andrejko et al., 2013) was utilized to demonstrate total extracellular protease production. Here, 100 μl SN was added to 100 μl of Azocasein (Sigma-Aldrich) solution (5 mg/ml in 0.1 M Tris–HCL, pH 8.8) and incubated at 37°C for 3 h. The reaction was stopped with 10% (v/v) trichloroacetic acid (25 μl per tube) and samples were centrifuged at 14,000 × *g* for 15 min at RT. To each well of a 96-well plate, 50 μl of 0.5 M NaOH was added to 50 μl of Azocasein SN in triplicate. NaOH was used as a blank, bacterial protease (Sigma-Aldrich) was included as a positive control, and absorbance was measured at 405 nm. For the Azocasein assay, one protease activity unit was defined as an absorbance increase (OD_405 nm_) of 0.02 per hour (Andrejko et al., 2013).

#### Virulence Factor Gene Expression

Samples snap frozen in liquid nitrogen were cut into <1 mm pieces, added to 1 ml Invitrogen^TM^ RNAlater^TM^ Stabilization Solution (Thermo Fisher Scientific) and 5 ml sterile borosilicate glass beads. Biofilm aggregates were dissociated from porcine skin as above and SN was centrifuged at 4°C to pellet bacterial cells. *P. aeruginosa* cell pellets were re-suspended in Max Bacterial Enhancement Reagent (Thermo Fisher Scientific) following the manufacturer’s protocol. RNA was then isolated with TRIzol^®^ reagent (Invitrogen, Thermo Fisher Scientific) and the aqueous phase purified with the PureLink RNA Mini Kit (Invitrogen, Thermo Fisher Scientific) following manufacturer’s instructions. RNA quantity was assessed using a Nanodrop spectrophotometer (Thermo Fisher Scientific), and reverse transcribed with Bioscript (Bioline). cDNA samples were diluted by three orders of magnitude and RT-qPCRs were carried out with Takyon mastermix (Eurogentec) on an IQ5 thermocycler (Bio-Rad Laboratories Ltd.). Relative expression was normalized using the housekeeping genes *16S* and *rpoD*. Primer sequences were as follows: *aprA*: sense 5′-CTTCAATACGCCGTGGAAGT-3′; antisense 5′-GCGTCGACGAAGTGGATATT-3′; *algD*: sense 5′-ATCAG CATCTTTGGTTTGGG-3′; antisense 5′-CACCAATG ACTTCATGACCG-3′; *lasB*: sense 5′-GTCGCAGTACTACAAC GGCA-3′; antisense 5′-ATTGGCCAACAGGTAGAACG-3′; *16S*: sense 5′-GTGGAAAAGAGCTTCTGGCA-3′; antisense 5′-CTT CTCGACGATGATTTCCG-3′; and *rpoD*: sense 5′-GCGACG GTATTCGAACTTGT-3′; antisense 5′-CGAAGAAGGAAATGG TCGAG-3′.

### Host-Response

#### Masson’s Trichrome Staining

Porcine skin and wounds were created for assessment of tissue culture viability. Skin and wounds were prepared as above and samples were cultured in DMEM containing 10% (v/v) FBS, at 37°C and 5% CO_2_ for 48 h. Explants were then bisected at their midpoint and (a) embedded in OCT or (b) fixed in 4% (v/v) saline-buffered formalin, processed, and embedded in paraffin wax. Overall structural assessment was performed with a modified version of the Goldner (1938) Masson’s trichrome stain. Sections brought to dH_2_O were stained in Weigert’s hematoxylin and Biebrich-Scarlet fuchsin, differentiated in phosphotungstic–phosphomolybdic acid and counterstained with Aniline Blue (all reagents from Sigma-Aldrich). Sections were dehydrated in 100% ethanol and mounted in Pertex^®^ (CellPath). Brightfield images were taken as above.

#### Immunohistochemistry

OCT embedded biofilm wounds, sectioned at 10 μm, were blocked in appropriate serum and incubated in primary antibodies O/N at 4°C. The antibodies used were the early apoptosis marker, goat anti-caspase 3 (R&D Systems), and the cell-proliferation marker, mouse anti-Ki67 (Novocastra). Alexa Fluor conjugated secondary antibodies (Thermo Fisher Scientific) were used to detect antibody binding. Sections were counterstained in DAPI and mounted in Mowiol 4-88 with DABCO (Thermo Fisher Scientific). Fluorescent images were taken as above using the DAPI, FITC and TEXAS RED filters.

#### Terminal Deoxynucleotidyl Transferase dUTP Nick End Labeling

An In Situ Cell Death Detection Kit (Roche) was used for “TUNEL” staining according to manufacturer’s instructions, using Proteinase K (Thermo Fisher Scientific) antigen retrieval prior to enzyme incubation. Fluorescent images were taken as above.

#### Picrosirius Red Staining

OCT sections were stained with Picrosirius Red to assess host matrix damage. Here, red birefringence indicated the presence of type I collagen, while yellow-green birefringence demonstrated the presence of type III collagen (Junqueira et al., 1979). Methanol-fixed sections were incubated in Picrosirius Red solution (0.5 g Sirius Red in 500 ml saturated picric acid, Sigma-Aldrich) for 1 h at RT, differentiated in 0.5% (v/v) acetic acid, dehydrated in ethanol, cleared in xylene and mounted in Pertex^®^ (CellPath). Polarized light images were taken on a Nikon E400 microscope with SPOT camera (SPOT imaging) using a polarizing filter set (Thermo Fisher Scientific). Analysis of collagen fiber composition was performed in ImageJ v.1.6 (NIH).

### Statistical Analyses

Data are expressed as average values ± standard deviations of the mean. *P*-values of less than 0.05 were considered significant. All statistical tests were performed in R v.3.3.3 (R Development Core Team, 2017) including the *car* (Fox and Weisberg, 2011) package. *T*-tests and one-way ANOVAs were completed, followed by Tukey’s HSD (honest significant differences) *post hoc* tests where applicable.

## Results

### Silver Bioactive Glass (Bg^Ag^) Elicited Potent Antimicrobial Efficacy Against Planktonic Cultures of *P. aeruginosa* and *S. aureus*

**FIGURE 1:**
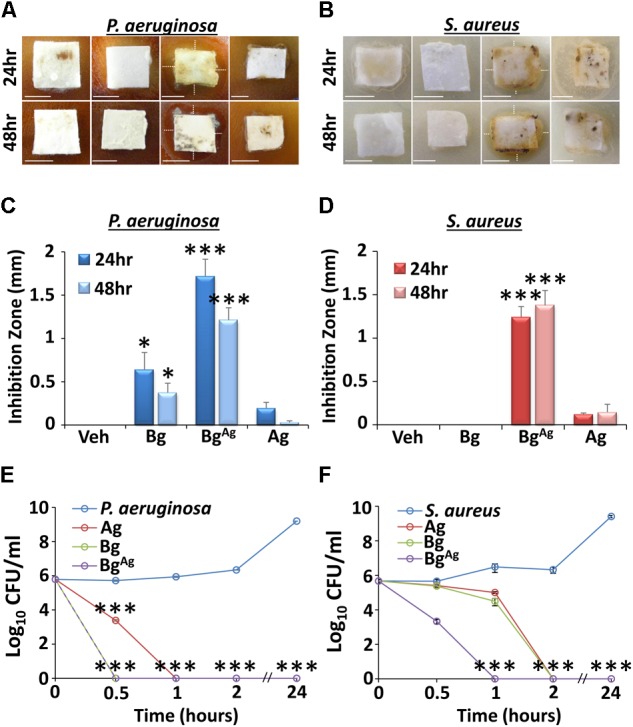
Planktonic agar diffusion **(A–D)** and time-kill **(E,F)** assays reveal differential antimicrobial effects of bioactive glass against *P. aeruginosa* **(A,C,E)** and *S. aureus* **(B,D,F)**. Inhibition zones (depicted by white, dotted lines) of agar diffusion assays were significantly greater in the Bg incorporating silver (Bg^Ag^) group compared to treatment with Bg alone (Bg), or a silver control (Ag). This effect was observed at both 24 and 48 h post-treatment, against *P. aeruginosa* **(A,C)** and *S. aureus* **(B,D)**, respectively. Interestingly, Bg significantly inhibited *P. aeruginosa* growth, but had no effect against *S. aureus* at both 24 and 48 h. As expected, the vehicle (Veh) created no inhibition zones against *P. aeruginosa* or *S. aureus*. Time-kill assays further illustrated disparate effects of Bg and Bg^Ag^ against *P. aeruginosa* and *S. aureus*. Here, Bg^Ag^ caused a 6-log reduction in *P. aeruginosa* **(E)** and *S. aureus* **(F)** within 30 min and 1 h, respectively. Data show the mean ± SEM. ^∗^*P* < 0.05 and ^∗∗∗^*P* < 0.001. Scale bars = 5 mm. Data were obtained from three independent experiments (*n* = 3 per treatment).

Agar diffusion assays with planktonic bacteria revealed differential effects for non-silver functionalized bioactive glass (Bg) and AgO-functionalized bioactive glass (Bg^Ag^) against *P. aeruginosa* and *S. aureus* (**Figures 1A–D**). Interestingly, Bg, which was moderately effective at inhibiting *P. aeruginosa* growth, displayed no inhibitory effect on *S. aureus*. By contrast, Bg^Ag^ elicited potent antimicrobial efficacy against both *P. aeruginosa* and *S. aureus* following 24- or 48-h treatments. The silver treatment alone (Ag), at equimolar concentration to the silver oxide component of Bg^Ag^, exhibited only marginal (non-significant) antimicrobial efficacy. Next, time-kill assays were used to define the temporal antimicrobial efficacy of Bg, Bg^Ag^, and Ag. In this assay, all treatments were potently antimicrobial (statistically significant 6-log reduction within 2 h). However, interesting species- and treatment-specific differences were observed. Both Bg and Bg^Ag^ led to a 6-log reduction in *P. aeruginosa* within 30 min, whereas this level of reduction took 60 min for Ag treatment (**Figure 1E**). *S. aureus* experiments revealed even greater differential treatment effects. Here, Bg^Ag^ alone led to a 6-log reduction in *S. aureus* within 1 h, while Bg and Ag took 2 h to achieve similar antimicrobial efficacy (**Figure 1F**). In summary, both agar diffusion and time-kill assays revealed both Bg and Bg^Ag^ to be potently antimicrobial against *P. aeruginosa*, while Bg^Ag^ maintained greater antimicrobial efficacy against *S. aureus* than Bg or Ag alone.

### Bg^Ag^, but Not Bg, Displays Maintained Antimicrobial Efficacy Over a Clinically Meaningful Time Frame

**FIGURE 2:**
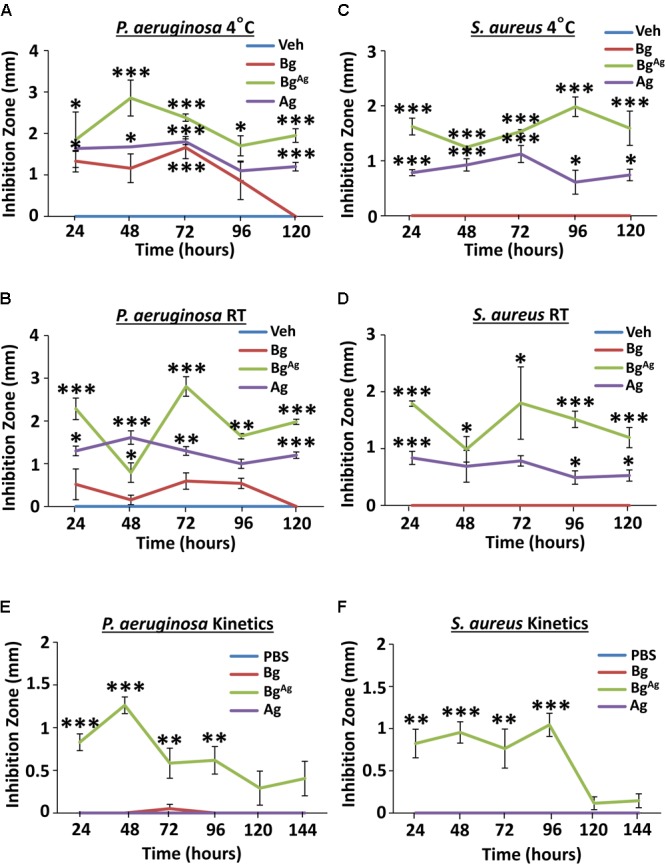
Bioactive glass incorporating silver maintains stability and releases soluble antimicrobial factors over a clinically relevant time frame. Bg, Ag, and Bg^Ag^, pre-incubated at 4°C **(A)** and RT **(B)** prior to 24-h agar diffusion assays, showed differential antimicrobial efficacy against *P. aeruginosa*. Bg^Ag^ and Ag caused significant *S. aureus* growth inhibition at 4°C **(C)** and RT **(D)**. Kinetic testing revealed release of soluble antimicrobial factors from Bg^Ag^, with potent antimicrobial effectiveness against *P. aeruginosa* **(E)** and *S. aureus* **(F)**. Points show the mean ± SEM. ^∗^*P* < 0.05, ^∗∗^*P* < 0.01, and ^∗∗∗^*P* < 0.001. Data were obtained from three independent experiments (*n* = 3 per treatment).

Agar diffusion assays were conducted using treatments that had been incubated at 4°C or RT for 24–120 h prior to assay (**Figure 2**). When newly constituted Bg^Ag^ and Ag were pre-incubated at 4°C, significant efficacy against *P. aeruginosa* was maintained over a period of 120 h. Bg on the other hand, maintained efficacy for only 72 h at 4°C (**Figure 2A**). Switching to RT, both Bg^Ag^ and Ag maintained potent efficacy against *P. aer* over a period of 120 h. By contrast, Bg displayed little inhibition at any time point (**Figure 2B**). In keeping with previous agar diffusion results (**Figure 1**), no *S. aureus* growth inhibition was observed following treatment with pre-incubated Bg at 4°C or RT (**Figures 2C,D**). By contrast Bg^Ag^ and Ag maintained efficacy against *S. aureus* over 120 h at both 4°C and RT (**Figures 2C,D**). An alternative measure related to treatment efficacy is the capacity to release antimicrobial over time. Here, the kinetics of release was measured by serial incubation of Bg, Ag, or Bg^Ag^ in PBS over six consecutive periods of 24 h. Here, Bg and Ag showed no antimicrobial efficacy against *P. aeruginosa* or *S. aureus* at any time point (**Figures 2E,F**). Bg^Ag^, however, strongly inhibited the growth of *P. aeruginosa* and *S. aureus* for up to 96 h (**Figures 2E,F**). Collectively, these data show that both Ag and Bg^Ag^ are highly stable, while Bg^Ag^ alone is able to release soluble antimicrobial factors over a 4-day time frame.

### Biofilm Formation Was Impaired by BG Treatment, With Differential Effectiveness Against *P. aeruginosa, S. aureus*, and Co-cultured Bacteria

Given the potent effects of Bg and Bg^Ag^ against planktonic bacteria, we next asked whether Bg or Bg^Ag^ were able to inhibit the formation of biofilms (microtiter method at OD_495 nm_; **Table 1**). In the absence of treatment, *P. aeruginosa* was considerably more effective at forming biofilm than *S. aureus*, or co-cultured bacteria (**Table 2**), which reflects previous studies (Bose et al., 2012; Nguyen and Oglesby-Sherrouse, 2016). *S. aureus* biofilm formation was entirely inhibited by 0.625% Bg^Ag^, while Bg treatment at the same concentration had no effect on *S. aureus* biofilm formation. For *P. aeruginosa*, both Bg and Bg^Ag^ strongly inhibited biofilm formation at higher concentration (0.625%). However, at the lower 0.15%, only Bg^Ag^ was able to partially inhibit biofilm formation. These results were recapitulated in co-culture, where 0.625% Bg had little effect on biofilm formation, while 0.625% Bg^Ag^ strongly inhibited biofilm formation. Taken together these data reveal greater anti-biofilm efficacy for Bg^Ag^ than Bg alone.

**Table 2:** The effects of bioactive glass (Bg) and Bg with silver (Bg^Ag^) on single-species and co-culture biofilm formation, based on OD measurements at 495 nm.

Treatment	*S. aureus* NCTC 13297			*P. aeruginosa* NCTC 10781			Co-culture		
	Concentration	Mean OD	Biofilm	Concentration	Mean OD	Biofilm	Concentration	Mean OD	Biofilm
Bacteria		0.1	Moderate		0.54	Strong		0.14	Moderate
Bg^Ag^	0.625%	0.04	Non-adherent	0.625%	0.08	Weak	0.625%	0.06	Weak
	0.15%	0.1	Moderate	0.15%	0.23	Strong	0.15%	0.09	Moderate
Bg	0.625%	0.11	Moderate	0.625%	0.07	Weak	0.625%	0.09	Moderate
	0.15%	0.1	Moderate	0.15%	0.5	Strong	0.15%	0.11	Moderate

### Bg^Ag^ Reduces Both *P. aeruginosa* and *S. aureus* Biofilms in an *ex Vivo* Porcine Wound Model

**FIGURE 3:**
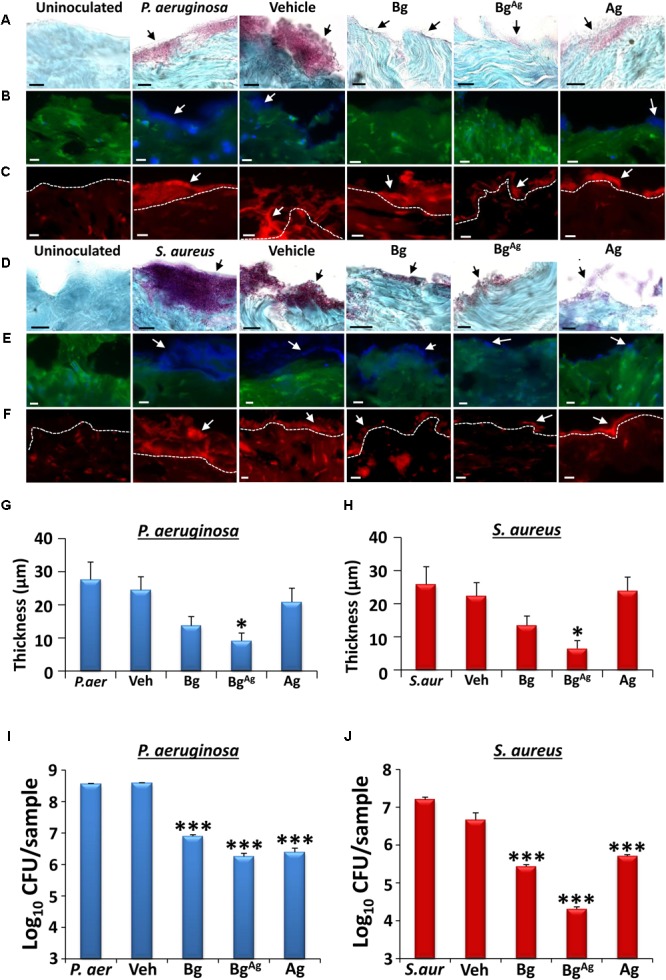
Bioactive glass reduces biofilm load and bacterial viability in an *ex vivo* porcine wound biofilm model. Modified Gram-Twort staining illustrated a reduction in *P. aeruginosa* (*P. aer*; pink; **A**) and *S. aureus* (*S. aur*; purple; **D**) biofilm load (black arrows), on porcine skin (blue) following Bg, Bg^Ag^ and Ag treatment. Quantification of Gram-Twort biofilm thickness (*n* = 9 images per treatment) showed a significant reduction in *P. aeruginosa* **(G)** and *S. aureus* **(H)** following Bg^Ag^ treatment only. Similarly, Concanavalin A (blue biofilm, **B,E**) and Acridine Orange (red biofilm, **C,F**) staining demonstrated decreased *P. aeruginosa* **(B,C)** and *S. aureus* **(E,F)** biofilm (depicted by white arrows) following bioactive glass treatment. Biofilm was not apparent on uninoculated porcine wounds. Bg, Bg^Ag^ and Ag also significantly reduced *P. aeruginosa* **(I)** and *S. aureus* **(J)** porcine biofilm load (CFU/ml) within 24 h of treatment (*n* = 3 biopsies per treatment). Black scale bars = 10 μm, white scale bars = 20 μm. Bars show mean ± SEM. ^∗^*P* < 0.05 and ^∗∗∗^*P* < 0.001.

To more faithfully replicate the *in vivo* environment, we turned to a combined *ex vivo* porcine skin/established bacterial biofilm model. Here, viable porcine wound explants were cultured following apical surface inoculation with established *P. aeruginosa* or *S. aureus* biofilms (**Figure 3**). Treatment for 24 h with Bg, Bg^Ag^, or Ag alone led to a visible reduction in biofilm thickness. Representative images illustrate biofilms visualized using Gram-Twort (*P. aeruginosa*, pink; *S. aureus*, purple; **Figures 3A,D**), ConA (**Figures 3B,E**), and AO (**Figures 3C,F**). Quantification of biofilm thickness from Gram-Twort stained sections across multiple replicates revealed that Bg^Ag^ treatment alone led to a statistically significant reduction in biofilm thickness (**Figures 3G,H**). Post-treatment viable colony enumeration (CFU/ml) was performed in parallel on dissociated biofilms. Here, all three treatments significantly reduced the number of viable biofilm bacteria (*P. aeruginosa* and *S. aureus* biofilms; **Figures 3I,J**), with Bg^Ag^ showing greatest efficacy against both bacterial species. These data directly demonstrate that silver-functionalized bioactive glass is effective against established *ex vivo* biofilms.

### Bg, Bg^Ag^, and Ag Treatments Differentially Influence *P. aeruginosa* Biofilm Virulence Factor Production

Bacterial colony enumeration alone does not directly convey bacterial pathogenicity (reviewed in Kallstrom, 2014), and effective invasion and adherence are fundamental requirements for establishing wound tissue infection (reviewed in Percival and McCarty, 2015). Thus, biofilm virulence was investigated via quantitative evaluation of extracellular protease activity and analysis of candidate virulence gene expression. In *P. aeruginosa*, these virulence factors include the endoproteases elastase (LasB) and alkaline protease (AprA), and alginate (AlgD; Goodman and Lory, 2004; Chu et al., 2016).

**FIGURE 4:**
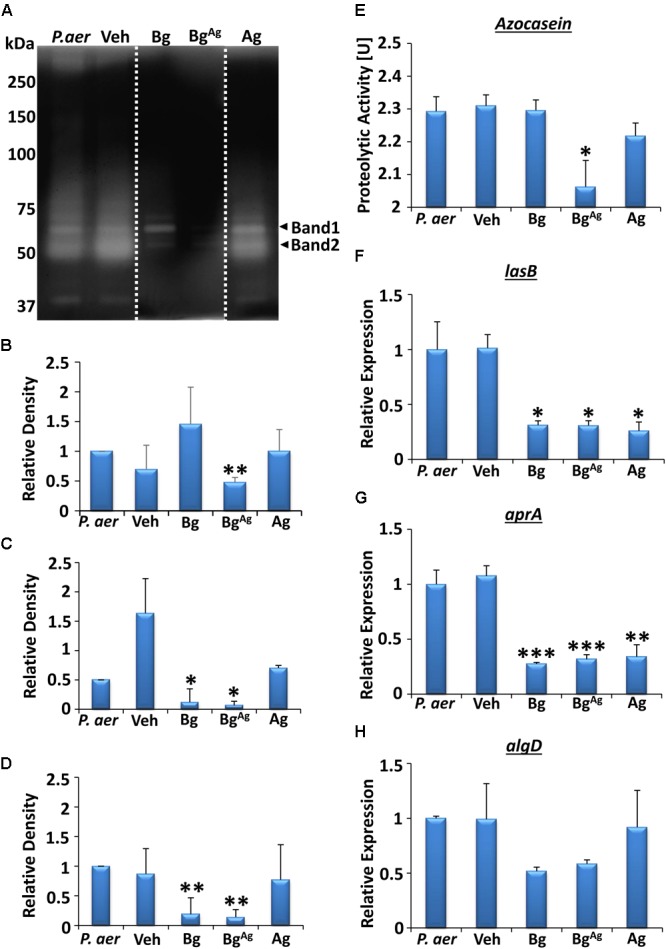
Extracellular protease activity from *P. aeruginosa* (*P. aer*) biofilms is differentially altered by bioactive glass treatment. Zymography analysis **(A–D)** illustrated changes in protease activity, where each lane showed *P. aeruginosa* biofilm (*P. aer*), vehicle (Veh), Bg, Bg^Ag^, and Ag treatments, respectively (lanes were cut and placed in this order, as shown via a white dotted line, representative of three gels). Significantly dampened activity of the protease in band 1 (∼70 kDa; **A,B**) was shown following Bg^Ag^ treatment, while both Bg and Bg^Ag^ significantly reduced protease activity in band 2 (∼50 kDa, **A,C**), and overall protease action **(D)**, compared to Veh. Colorimetric analysis (repeated in three independent experiments) of total extracellular protease **(E)** demonstrated a reduction in proteolytic activity with Bg^Ag^, compared to Veh. Significant reductions (compared to Veh) were also observed in expression of the virulence genes *lasB* **(F)** and *aprA* **(G)**, while no significant changes were shown in *algD* **(H)**, illustrated via RT-qPCR. Data represent the mean ± SEM. ^∗^*P* < 0.05, ^∗∗^*P* < 0.01, and ^∗∗∗^*P* < 0.001.

Zymography was performed to elucidate changes in bacterial protease activity (**Figure 4**). *P. aeruginosa* biofilm samples gave a range of bands on a gelatin zymogram (original gel, Supplementary Figure S1). Quantification was performed for the principle bands (∼70 and ∼50 kDa) in addition to total activity. Protease activity depicted by band 1 (∼70 kDa), described as a distinct version of elastase (Lomholt et al., 2001), was significantly inhibited by Bg^Ag^ compared to the vehicle (**Figures 4A,B**), but not Bg or Ag alone. A different pattern of activity, where both Bg and Bg^Ag^ reduced expression compared to the vehicle control, was observed for band 2 (∼50 kDa), which corresponds to the molecular mass of alkaline protease activity (Caballero et al., 2001; Lomholt et al., 2001; **Figures 4A,C**). Finally, when total protease activity within each lane was measured, Bg^Ag^ and Bg significantly inhibited protease activity (**Figures 4A,D**). Independent colorimetric protease analysis, using the Azocasein method (Andrejko et al., 2013) was performed to quantify total biofilm protease activity following treatment (**Figure 4E**). Here, total protease activity was significantly reduced following Bg^Ag^ treatment alone, compared to the vehicle.

We next switched to evaluating virulence factor gene expression in *P. aeruginosa* biofilms. Analysis revealed that all three treatments significantly reduced expression of the gene *lasB* compared to Veh (*P* < 0.05, **Figure 4F**), important in encoding elastase B production (Casilag et al., 2016). Similarly, Ag significantly reduced *aprA* expression (*P* < 0.01), a gene encoding alkaline protease (Lomholt et al., 2001; **Figure 4G**), while Bg and Bg^Ag^ treatment led to a further reduction in *aprA* expression (*P* < 0.001). Finally, reduced expression of *algD* (important for alginate biosynthesis; Wiens et al., 2014) was apparent following Bg and Bg^Ag^ treatment, although this failed to reach significance (**Figure 4H**).

### Bg^Ag^ Protects Porcine Tissue From Biofilm Induced Dermal Cell Death and ECM Turnover

**FIGURE 5:**
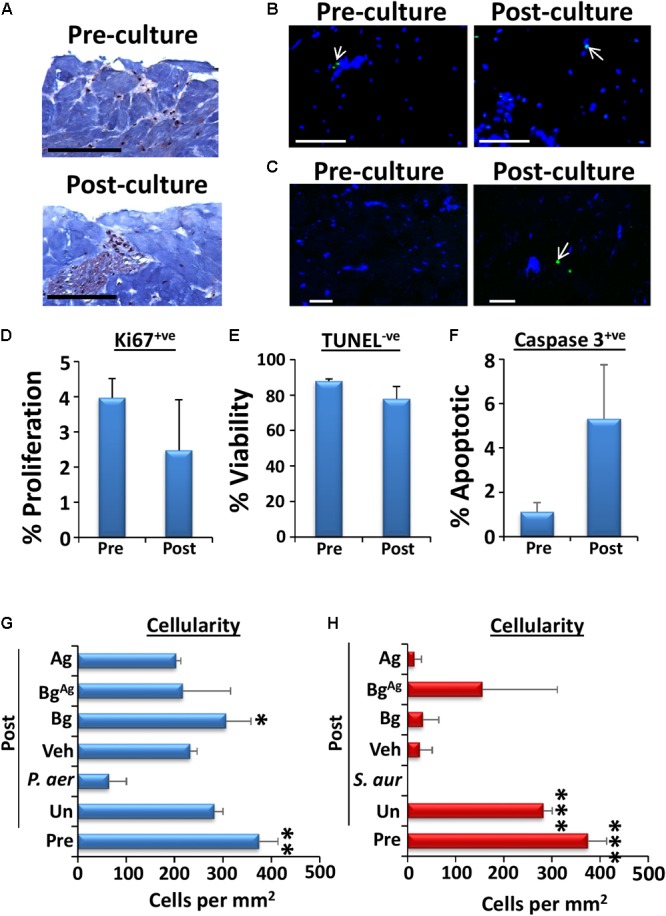
Viability of porcine explants is drastically reduced by biofilm treatment, yet Bg^Ag^ prevents decellularization. Viability of uninoculated porcine tissue over the 48-h culture period was assessed via comparison of wounds pre-culture (“Pre”) and post-culture (“Post”) at 37°C and 5% CO_2_ (*n* = 3 per group). Viability was deduced via visualization of structure (**A**, Masson’s trichrome staining), cell proliferation (Ki67^+ve^ staining, **D**), cell viability (TUNEL^-ve^ cells, blue, **B** and **E**) and apoptosis (caspase 3^+ve^ cells, green, **C** and **F**). Cell death following *P. aeruginosa* (*P. aer*, **G**) and *S. aureus* (*S. aur*, **H**) biofilm loading was assessed by measuring cellularity (number of DAPI stained cells per mm^2^) post-culture (48 h, *n* = 9 images per group). Cellularity of pre-culture porcine tissue was also assessed. Arrows depict positively stained green cells. Black bars = 100 μm, white bars = 20 μm. White arrows depict green (apoptotic) cells. ^∗^*P* < 0.05, ^∗∗^ *P* < 0.01, ^∗∗∗^*P* < 0.001.

**FIGURE 6:**
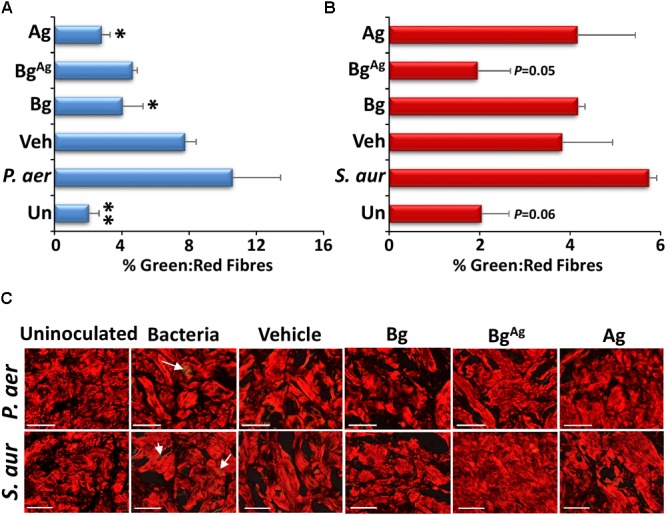
Bioactive glass protects against extracellular matrix turnover. Picrosirius red staining of porcine biofilm tissue (*n* = 9 per group) demonstrated changes in collagen III turnover via measurement of % green: red fibers (green birefringence is demonstrated by white arrows). Significantly more collagen III turnover was demonstrated in *P. aeruginosa* (*P. aer*, **A,C**) biofilms compared to uninoculated controls, Bg and Ag treatments. *S. aureus* (*S. aur*, **B,C**) biofilms increased collagen III turnover compared to Bg^Ag^ and uninoculated porcine explants. ^∗^*P* < 0.05, ^∗∗^*P* < 0.01. White bars = 100 μm.

We next switched our attention to assessing the direct effects of bacterial biofilm exposure on porcine wound tissue. Initial histological evaluation of non-biofilm infected skin revealed comparable levels of cell proliferation prior to culture (4%) versus after 48 h of *ex vivo* culture (3%; **Figure 5D**), agreeing with previously published data (Yeung et al., 2016). Similarly, only a marginal increase in cell death was observed following 48 h of *ex vivo* culture, with around 20% of cells TUNEL^+ve^ (**Figures 5B,E**) and around 5% of cells undergoing apoptosis (caspase 3^+ve^; **Figures 5C,F**). In stark contrast, skin that was maintained *ex vivo* for 48 h in the presence of biofilm displayed significantly high levels of decellularity compared to pre-cultured tissue (**Figures 5G,H**). Notably, Bg treatment significantly reduced this biofilm-induced cell lysis in *P. aeruginosa* inoculated wounds (**Figure 5G**), while Bg^Ag^ retained increased cellularity compared to biofilm controls. Finally, given the effects of bioactive glass treatment on bacterial protease production (**Figure 4**), we asked whether changes in skin extracellular matrix composition would be evident. Interestingly, biofilm infection increased collagen turnover at the biofilm skin interface [visualized by an increase in new (green birefringence) fiber production; **Figure 6**]. Again, treatment with Bg, Bg^Ag^, and Ag decreased this turnover in wounds inoculated with *P. aeruginosa* biofilms, reducing the amount of new matrix produced (**Figures 6A,C**). Interestingly, in *S. aureus* wounds, Bg^Ag^ treatment alone maintained a protective effect against collagen turnover (**Figures 6B,C**). Thus, Bg treatment is not only effective at killing biofilm bacteria, but it also demonstrably reduces the direct detrimental effects of bacteria on the host tissue.

### Disparate Anti-biofilm Effects of BG on *P. aeruginosa*: *S. aureus* Co-culture Porcine Wound Biofilms

**FIGURE 7:**
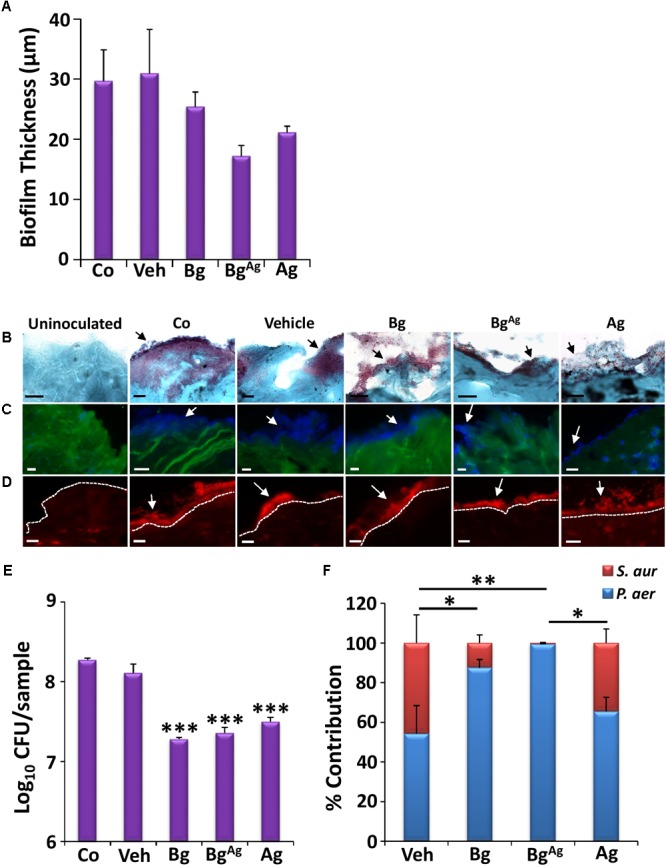
Co-culture biofilms of *P. aeruginosa* (*P. aer*) and *S. aureus* (*S. aur*) appear more resistant to bioactive glass than single-species biofilms. Quantification of biofilm thickness (**A**; *n* = 9 images per group) via Gram-Twort staining **(B)**, revealed no significant change in biofilm load (black arrows) with bioactive glass treatment. Further visualization with Concanavalin A (**C**, blue biofilms), and Acridine Orange (**D**, red biofilms, above the white-dotted line) confirmed this, where white arrows illustrate biofilms. However, Bg, silver Bg (Bg^Ag^), and silver alone (Ag) caused a significant reduction in porcine biofilm load (CFU/ml) within 24 h **(E)**. The percentage of *P. aeruginosa* and *S. aureus* contributing to the co-culture porcine biofilms was also determined following treatment, where statistical analysis shows significant changes in the contribution of *P. aeruginosa* and *S. aureus* between treatments **(F)**. Black scale bars = 10 μm, white scale bars = 20 μm. Bars show the mean ± SEM. ^∗^*P* < 0.05, ^∗∗^*P* < 0.01, and ^∗∗∗^*P* < 0.001.

The prevalence of polymicrobial infections is a major clinical problem, which contributes to the intransigent nature of chronic wounds (Pastar et al., 2013; Fleming et al., 2017). To partially mimic this phenomenon, we established a co-culture porcine wound biofilm model. Interestingly, all three treatments were less effective at killing bacteria in co-cultured biofilms than in single culture biofilms (**Figure 3**). These effects were confirmed by direct visualization of the co-culture biofilms, where Bg, Bg^Ag^, and Ag treatment led to no more than a mild qualitative reduction in wound biofilm load via Gram-Twort (**Figure 7B**), ConA (**Figure 7C**), and AO staining (**Figure 7D**). Quantification of biofilm thickness revealed only a non-significant trend toward reduced biofilm thickness following treatments (**Figure 7A**). We did, however, observe a statistically significant reduction in total CFU following Bg, Bg^Ag^, and Ag treatment of co-cultured biofilms (**Figure 7E**). Overall, these data demonstrated a less pronounced effect of Bg and Bg^Ag^ against co-culture biofilms than single-species biofilms.

More clinically significant was the effect of treatments on the relative proportion of *P. aeruginosa* and *S. aureus* in co-cultured biofilms (**Figure 7F**). At the time of collection (48 h post tissue inoculation), vehicle-treated biofilms were quantified as 55:45 *P. aeruginosa* to *S. aureus* relative proportions. Ag treatment led to a modest non-significant shift in these proportions to around 65:35. By contrast, Bg treatment led to a large, statistically significant shift to almost 90:10 (*P* < 0.05, in both *P. aeruginosa* and *S. aureus* contribution). The greatest effect was observed following Bg^Ag^ treatment, where virtually no *S. aureus* could be recovered (*P* < 0.01, in *P. aeruginosa* and *S. aureus*). These data, which mirror the differential selectivity of BG treatment against single-species biofilms (**Figure 3**), reveal the extent to which a topical wound treatment can alter the dynamic equilibrium between two bacterial species.

## Discussion

Though it is axiomatic that silver has potent antimicrobial effects against a range of bacterial species (reviewed in Maillard and Hartemann, 2013), comparatively few studies have addressed the antimicrobial and anti-biofilm efficacy of BG formulations. Fewer still have explored BG formulations functionalized with silver (e.g., Drago et al., 2014; Gholipourmalekabadi et al., 2016). Here, we assessed the bactericidal activity of BG against planktonic and biofilm forms of wound-relevant bacteria (*P. aeruginosa* and *S. aureus*; James et al., 2008). Our data clearly show that BG incorporating silver (Bg^Ag^) provided a more potent bactericidal effect against planktonic *P. aeruginosa* and *S. aureus* than Bg or Ag alone. Previously, Ag treatment has been shown to increase membrane permeability and potentiate the susceptibility of Gram-negative bacteria to antibiotics (Morones-Ramirez et al., 2013). Therefore, in the present work, Ag may also be potentiating microbial susceptibility to bioactive glass. An important novel observation was the relative species-specific efficacy, with Bg more effective against planktonic *P. aeruginosa* than *S. aureus* (**Figure 1**). In agar diffusion tests, Bg alone failed to cause inhibition of *S. aureus* growth. One previously suggested reason for this is that bacterial metabolism (acid production) may circumvent increased pH, thus reduce the diffusibility of Bg into agar (Stoor et al., 1998; Martins et al., 2011). However, the observed differential effects of Bg most likely reflect the differences in cell-wall structure between the two bacterial species. The Gram-positive *S. aureus* is encapsulated by a thickened peptidoglycan cell wall which has been suggested to convey increased resistance (Zoeiby et al., 2001). Indeed, a number of *S. aureus* strains maintain antibiotic-tolerance via cell wall-related mechanisms (Utaida et al., 2003; Dengler et al., 2011). The less substantial *P. aeruginosa* cell wall and membrane structure may confer susceptibility to the increased pH caused by Bg ion dissolution (e.g., [Ca^2+^]i and [Na^+^]i release; Stoor et al., 1998), thus altering bacterial membrane potential (Munukka et al., 2008) and causing osmotic stress (Van der Waal et al., 2011). Furthermore, regional variation in skin pH is thought to play a significant role in spatial localisation of skin bacteria (reviewed in Schreml et al., 2010), while *S. aureus* is known to favor more acidic conditions than *P. aeruginosa* (Ushijima et al., 1984). In the present study, the effects of BG may also be due to the antimicrobial efficacy of its relative components, e.g., SiO_2_ (Maçon et al., 2017; Siqueira et al., 2017).

Of more physiological relevance, BG significantly reduced *P. aeruginosa* and *S. aureus* biofilm load in an *ex vivo* porcine wound explant model, albeit less effectively than during *in vitro* planktonic assays (**Figure 3**). Curiously, species-specific efficacy was reversed in this biofilm model, with *S. aureus* more susceptible to BG treatment than *P. aeruginosa.* This may simply reflect the strong biofilm-producing ability of *P. aeruginosa* (Harrison-Balestra et al., 2003) accompanied by production of a multitude of extracellular proteases (Andrejko et al., 2013). By contrast, *S. aureus* forms weak biofilms initially, relying on adhesion proteins and autolysin production for its virulence and biofilm generation (Rice et al., 2007; Bose et al., 2012; Foulston et al., 2014). *S. aureus* favors acidic conditions in the clinical setting (Weinrick et al., 2004), and a well characterized drop in pH triggers *S. aureus* biofilm matrix production and subsequent cell aggregation. As BG treatment increases pH (∼10 in the present study, data not shown), we postulate that alkaline stress caused by BG could contribute to reduced biofilm load. Here the literature is conflicting with respect to *S. aureus*; Foulston et al. (2014) report reduced biofilm formation at high pH, while Van der Waal et al. (2011) report no effect on biofilm viability.

The most common method for determining bacterial load in samples is through assessing colony viability (Buysschaert et al., 2016). However, recent evidence suggests some bacteria, including *P. aeruginosa* and *S. aureus*, can enter a viable but not culturable (VBNC) state, where they may contribute to biofilm virulence (Zhang et al., 2015). Alterations in exogenous stimuli are known to modify *P. aeruginosa* virulence factor expression (Rumbaugh et al., 1999; Shigematsu et al., 2001; Breidenstein et al., 2011), largely through quorum-sensing systems (Dong and Zhang, 2005). In quantifying virulence factor expression, we focused on genes responsible for modulating protease production and bacterial cell–host matrix binding, fundamental requirements for establishing wound tissue infection (reviewed in Percival and McCarty, 2015). Bg, Bg^Ag^, and Ag significantly reduced expression of the *P. aeruginosa* virulence factors; elastase (*LasB*) and alkaline protease (*AprA*; Goodman and Lory, 2004; Lee et al., 2013; Khosravi et al., 2016). Direct analysis of *P. aeruginosa* gelatinases confirmed the effect of bioactive glass, where Bg and Bg^Ag^ demonstrated a substantial reduction in activity corresponding to elastase (∼37 kDa; Miyajima et al., 2001) and alkaline protease (50–75 kDa; Engel et al., 1997; Schmidtchen et al., 2003; **Figure 4**). Elastase (LasB) is particularly interesting as it exhibits potent widespread proteolytic activity, thus causing excessive wound tissue proteolysis (Kessler et al., 1998) while preventing normal dermal fibroblast growth (Schmidtchen et al., 2003). Indeed, when we assessed the effect of *P. aeruginosa* and *S. aureus* biofilms on host tissue we found reduced cellularity and increased matrix turnover (**Figures 5, 6**, respectively).

Chronic wounds are a term used to encompass all wounds that fail to heal within 12 weeks and can include, but are not limited to, diabetic foot ulcers, pressure sores, and venous leg ulcers (Rodriguez-Arguello et al., 2018). Chronic wounds often remain recalcitrant due to their diverse microbial communities (Gardner et al., 2013; Kalan et al., 2016), which can profoundly affect clinical outcome (Baldan et al., 2014; Wolcott et al., 2015). Although the consequences of interspecies interactions on wound pathogenesis remain to the elucidated, experimental studies have revealed that multiple-species biofilms exert major influence on host tissue responses, such as inflammation (Pastar et al., 2013). Therefore, we produced co-culture porcine biofilms of *S. aureus* and *P. aeruginosa* to further investigate the efficacy of BG treatment. Our experiments also revealed that co-cultured biofilms were more resistant to BG treatment, where only a modest reduction in viability was observed (**Figure 7**). Experimentally producing polymicrobial biofilm communities can be difficult as one species often predominates (Malic et al., 2009; Dalton et al., 2011). In our study, untreated porcine biofilms adopted a stable ratio at around 60:40 *P. aeruginosa* to *S. aureus*, in line with the potentially synergistic relationship between the two species (Dalton et al., 2011; Korgaonkar et al., 2013; Pastar et al., 2013). Curiously, following BG-treatment, *P. aeruginosa* quickly became dominant. This supports studies demonstrating that *P. aeruginosa* may out-compete *S. aureus* when resources are limited, in part by sequestering vital cofactors, and producing metabolites toxic to *S. aureus* (Mashburn et al., 2005; Biswas et al., 2009; DeLeon et al., 2014).

Collectively, this study demonstrates clear potential for Bg and Bg^Ag^ as potential wound-relevant antimicrobials. It is crucial that we continue to explore novel antimicrobial agents, especially for indications where mechanisms of antibiotic resistance and silver resistance are a concern (Su et al., 2011; Randall et al., 2015). This is particularly important when considering the polymicrobial interactions of clinical infections (Dalton et al., 2011). Although not addressed in the present study, BG presents an attractive opportunity for future functionalization with a variety of compounds designed to promote wound repair (Bonvallet et al., 2015; Wu et al., 2017). Combining this versatility with clear antimicrobial efficacy offers exciting opportunities for wound management.

## Author Contributions

HW and MH were involved in study design. HW and SI performed the experiments and analyzed the data. HW, SI, and MH interpreted the data. HW and MH wrote the manuscript. HW, SI, PC, and MH read and approved the final manuscript for publication.

## Conflict of Interest Statement

PC is the CEO of Theraglass^TM^ Ltd. The remaining authors declare that the research was conducted in the absence of any commercial or financial relationships that could be construed as a potential conflict of interest.
