# Supplementary figures and images for: A Novel Silver Bioactive Glass Elicits Antimicrobial Efficacy Against Pseudomonas aeruginosa and Staphylococcus aureus in an ex Vivo Skin Wound Biofilm Model
Source: Front Microbiol. 2018 Jul 3;9:1450. doi: 10.3389/fmicb.2018.01450 (PMC6037725; doi:10.3389/fmicb.2018.01450)

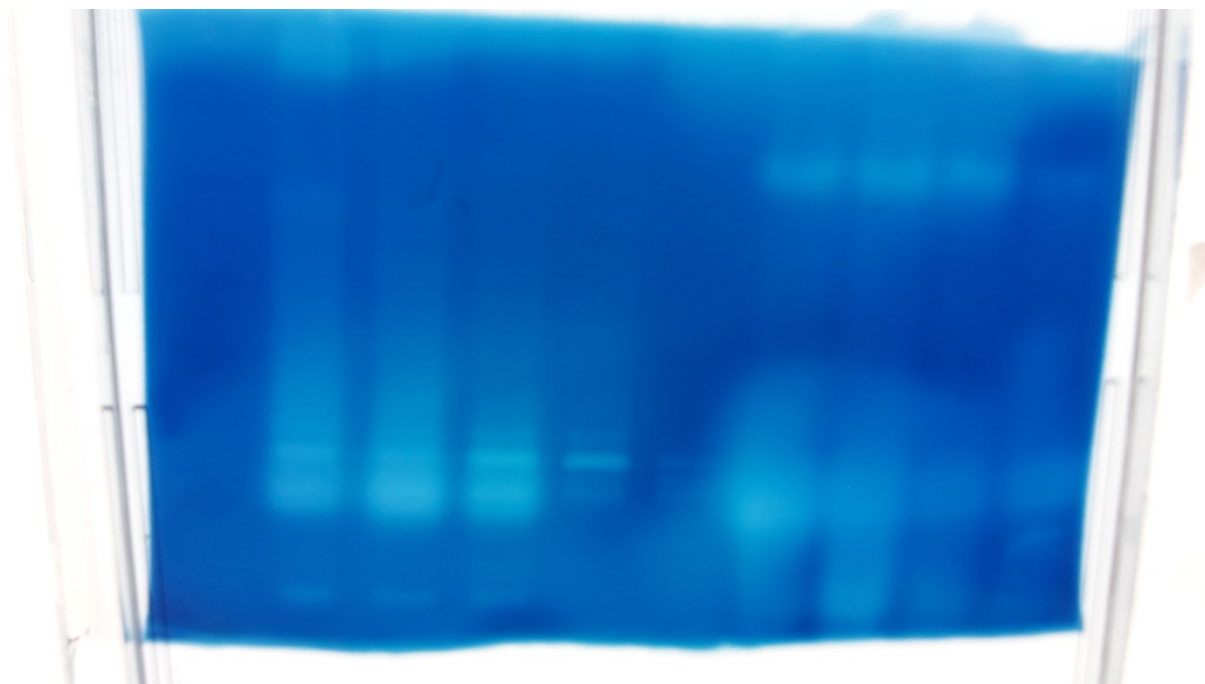

Figure S1. Original Zymogram for Figure 4.

Supplement: Supplementary file 1 [file Image_1.pdf]
